# Neurological Complications and Noninvasive Multimodal Neuromonitoring in Critically Ill Mechanically Ventilated COVID-19 Patients

**DOI:** 10.3389/fneur.2020.602114

**Published:** 2020-11-27

**Authors:** Denise Battaglini, Gregorio Santori, Karthikka Chandraptham, Francesca Iannuzzi, Matilde Bastianello, Fabio Tarantino, Lorenzo Ball, Daniele Roberto Giacobbe, Antonio Vena, Matteo Bassetti, Matilde Inglese, Antonio Uccelli, Patricia Rieken Macedo Rocco, Nicolò Patroniti, Iole Brunetti, Paolo Pelosi, Chiara Robba

**Affiliations:** ^1^Anesthesia and Intensive Care, San Martino Policlinico Hospital, Istituto di Ricovero e Cura a Carattere Scientifico for Oncology and Neurosciences, Genoa, Italy; ^2^Department of Medicine, University of Barcelona, Barcelona, Spain; ^3^Department of Surgical Sciences and Integrated Diagnostic (DISC), University of Genoa, Genoa, Italy; ^4^Department of Infectious Diseases, San Martino Policlinico Hospital - Istituto di Ricovero e Cura a Carattere Scientifico for Oncology and Neurosciences, Genoa, Italy; ^5^Department of Health Sciences (DISSAL), University of Genoa, Genoa, Italy; ^6^Department of Neurology, Rehabilitation, Ophthalmology, Genetics, Maternal and Child Health (DINOGMI), University of Genoa, Genoa, Italy; ^7^Laboratory of Pulmonary Investigation, Carlos Chagas Filho Institute of Biophysics, Federal University of Rio de Janeiro, Rio de Janeiro, Brazil; ^8^Rio de Janeiro Network on Neuroinflammation, Carlos Chagas Filho Foundation for Supporting Research in the State of Rio de Janeiro (FAPERJ), Rio de Janeiro, Brazil; ^9^COVID-19 Virus Network, Ministry of Science and Technology, and Innovation, Rio de Janeiro, Brazil

**Keywords:** COVID-19, neurological complications, SARS-CoV-2, neuromonitoring, neurocritical care

## Abstract

**Purpose:** The incidence and the clinical presentation of neurological manifestations of coronavirus disease-2019 (COVID-19) remain unclear. No data regarding the use of neuromonitoring tools in this group of patients are available.

**Methods:** This is a retrospective study of prospectively collected data. The primary aim was to assess the incidence and the type of neurological complications in critically ill COVID-19 patients and their effect on survival as well as on hospital and intensive care unit (ICU) length of stay. The secondary aim was to describe cerebral hemodynamic changes detected by noninvasive neuromonitoring modalities such as transcranial Doppler, optic nerve sheath diameter (ONSD), and automated pupillometry.

**Results:** Ninety-four patients with COVID-19 admitted to an ICU from February 28 to June 30, 2020, were included in this study. Fifty-three patients underwent noninvasive neuromonitoring. Neurological complications were detected in 50% of patients, with delirium as the most common manifestation. Patients with neurological complications, compared to those without, had longer hospital (36.8 ± 25.1 vs. 19.4 ± 16.9 days, *p* < 0.001) and ICU (31.5 ± 22.6 vs. 11.5±10.1 days, *p* < 0.001) stay. The duration of mechanical ventilation was independently associated with the risk of developing neurological complications (odds ratio 1.100, 95% CI 1.046–1.175, *p* = 0.001). Patients with increased intracranial pressure measured by ONSD (19% of the overall population) had longer ICU stay.

**Conclusions:** Neurological complications are common in critically ill patients with COVID-19 receiving invasive mechanical ventilation and are associated with prolonged ICU length of stay. Multimodal noninvasive neuromonitoring systems are useful tools for the early detection of variations in cerebrovascular parameters in COVID-19.

## Introduction

Coronavirus disease 2019 (COVID-19), caused by the severe acute respiratory syndrome coronavirus-2 (SARS-CoV-2) (1–4), is primarily a disease of the respiratory system, leading to a variety of clinical manifestations including dry cough, fever, fatigue, and respiratory failure (4). However, recent data suggest that COVID-19 is not confined to the airways but is also responsible for a syndrome of multiorgan dysfunction, including possible neurological involvement (5, 6).

Coronaviruses may pass to the central nervous system by different routes (7, 8), including hematogenous spread from the systemic to the cerebral circulation and lymphocyte invasion or dissemination from the cribriform plate and olfactory bulb to the brain (9, 10). These hypothesis seem to be consistent with the loss of smell and taste described as—first atypical, then quite prevalent—presentations of COVID-19 (11). However, the neurologic manifestations of COVID-19 are highly variable and can occur prior to diagnosis or as a complication late in the course of infection (7, 8).

A recent systematic review of 37 articles revealed that 20% of COVID-19 patients present with headache, 60% with anosmia/ageusia, 25% with myalgia/myositis, 8.8% with encephalopathy, 2.8% with ischemic stroke, and 0.45% with intracerebral hemorrhage (12). Other neurological symptoms include impaired consciousness, ataxia, seizures, and neuralgia (13–17). SARS-CoV-2 has also been reported to trigger autoimmune diseases such as multiple sclerosis, acute disseminated encephalomyelitis, acute encephalitis, acute autoimmune polyneuropathy, and critical illness polyneuropathy (13, 18) as well as cerebrovascular events (19, 20). However, recent reports suggest that hypoxic–ischemic damage could be the main driver of neurological symptoms in COVID-19 patients (21).

Noninvasive neuromonitoring systems are widely used in neurointensive care settings for patients with primary cerebral damage; more recently, they are also being employed in critically ill patients in general as useful tools to detect neurological complications (22, 23). In particular, transcranial Doppler (TCD) ultrasonography, optic nerve sheath diameter (ONSD) measurement, and quantitative automated pupillometry are safe, useful methods that can be applied at the patient's bedside to assess cerebral hemodynamics as well as to monitor cerebral perfusion pressure and intracranial pressure noninvasively (22, 23). To date, no studies have investigated cerebral hemodynamics in patients with COVID-19.

The primary aim of our study was to describe the type and the frequency of neurological complications in a cohort of critically ill patients with COVID-19 receiving invasive mechanical ventilation in an intensive care unit (ICU) and the effects of these complications on outcome. As a secondary aim, we sought to assess changes in cerebral hemodynamics, their effects on outcome, and their role as potential predictors of neurological complications in a subgroup of patients who underwent noninvasive neuromonitoring (ONSD, TCD, and automated pupillometry).

## Materials and Methods

### Study Design

This is a single-center, retrospective, observational study of prospectively collected data. The study was carried out during the COVID-19 pandemic, from February 28 through June 30, 2020, at the ICU of the San Martino Policlinico Hospital (SMPH) IRCCS for Oncology and Neurosciences, Genoa, Italy. The SMPH is the main hospital serving both the metropolitan area of Genoa (approximate population of 840,000) and the wider Liguria Region (approximate population of 1,543,000). The usual ICU capacity is 52 adult beds, increased to 74 during the peak of the SARS-CoV-2 outbreak in Italy. The study protocol followed good clinical practice principles in compliance with the Declaration of Helsinki, and the Ethics Committee of Liguria, Italy (registry number 163/2020), approved the study and waived the informed consent for participation because of the retrospective nature of the study.

### Study Population

Patients aged ≥18 years, confirmed positive for SARS-CoV-2 infection by reverse transcriptase-polymerase chain reaction (RT-PCR) of nasopharyngeal swab specimens at the moment of ICU admission, and who were critically ill, requiring invasive mechanical ventilation, were eligible for inclusion. Patients who were not neurologically evaluable due to deep sedation for life-threatening respiratory failure were excluded.

### Data Collection

#### Overall Population

The following data were collected from the patients' electronic records at the time of ICU admission: age in years, gender, body mass index (in kg/m^2^), sequential organ failure assessment score (24), and a series of comorbidities, namely, hypertension, diabetes mellitus, respiratory disease (defined as asthma or chronic obstructive pulmonary disease), end-stage renal disease (defined as estimated glomerular filtration rate <15 ml/min/1.73 m^2^), moderate/severe liver disease (defined as compensated/decompensated liver cirrhosis) (25), and cancer. The highest C-reactive protein (normal range 0–5 mg/L) and D-dimer (normal range 0–500 mcg/L) as well as the lowest partial pressure of oxygen (PaO_2_) (normal range 72–104 mmHg) were collected from daily test results throughout each patient's ICU stay. At the time of ICU and hospital discharge, data on ICU length of stay (ICU-LOS) (days), overall hospital LOS (days), duration of mechanical ventilation (days), neurological complications (type and number), and mortality were collected.

#### Neuromonitoring Cohort

The following data were collected from patients who underwent noninvasive neuromonitoring during the day of assessment and throughout their ICU stay: ventilatory parameters [type of ventilation, positive end-expiratory pressure (PEEP) in cmH_2_O, pressure control or pressure support in cmH_2_O, respiratory rate in breaths per minute, tidal volume in mL, and fraction of inspired oxygen (FiO_2_)], arterial blood gas values [PaO_2_ in mmHg, partial pressure of carbon dioxide (PaCO_2_) in mmHg, pH], vital signs [mean arterial pressure (MAP) in mmHg, heart rate in beats per minute], sedation (including type of sedative), analgesia (including analgesic agent), and neuromuscular blockade. The neurological complications and scales used for outcome measures are defined in the Supplementary Tables 1–4.

### Noninvasive Neuromonitoring Systems

Ultrasound measurements were performed by two experienced operators (defined as having received more than 5 years of training and performing more than 70 examinations/year) (DB, CR) and three mentored trainees in anesthesia and intensive care (KC, FI, MB). MAP, heart rate, mean cerebral artery (MCA) flow velocities (diastolic, mean, and systolic), and ONSD were recorded during ICU stay, according to the clinical context and need (availability of personal protective equipment and clinical rationale).

#### Transcranial Doppler

A low-frequency (2 MHz) microconvex transducer (Philips SparQ®) was used to investigate intracranial vessels. The temporal window was preferred for passage of the Doppler signal for MCA assessment. Systolic (sFV), diastolic (dFV), and mean flow velocity (mFV) in the MCA were collected. MAP was also measured. The pulsatility index (PI) was calculated as the mean value between the right and the left MCA flow velocities using the following formula (13):

$$\begin{array}{l} PI=\frac{\left(sFV-dFV\right)}{mFV} \end{array}$$

Noninvasive ICP (nICP_TCD_) was calculated according to the formula:

$$\begin{array}{l} nICP_{\text{TCD}}=MAP-CPPe \end{array}$$

where cerebral perfusion pressure (CPPe) was calculated as follows (26):

$$\begin{array}{l} CPPe=\frac{MAP\;*dFV}{mFV}+14 \end{array}$$

Intracranial pressure (ICP) values >20 mmHg were considered indicative of intracranial hypertension (26).

#### Optic Nerve Sheath Diameter

A linear probe (Philips SparQ®) was used for ONSD evaluation. The probe was placed on the closed upper eyelid, and ONSD was evaluated 3 mm behind the retinal papilla. Two measurements were obtained from each optic nerve: the first in the transverse plane and the second in the sagittal plane (27). Noninvasive intracranial pressure measured by ONSD (nICP_ONSD_) was derived from a mathematic formula described elsewhere in the literature (28, 29). ICP values >20 mmHg were again considered indicative of intracranial hypertension (26).

#### Automated Pupillometry

Pupillary light reactivity was measured by a handheld quantitative automated pupillometer (Neurolight Algiscan®, ID-MED, Marseille, France) in both eyes. This device measures quantitative variation in pupillary light reactivity by using an infrared camera to record a video footage of the changes in the pupillary surface. Pupillary light reactivity was assessed by a calibrated light stimulation (320 lux for 1 s) with a precision limit of 0.05 mm. Quantitative reactivity was expressed as the percentage of pupillary light response, and baseline pupil size was expressed in millimeters. The pupillary constriction velocity (mm/s) was also reported (30–32). Abnormal pupillary reactivity was defined as an abnormal pupillary light reflex as reported by the pupillometer (e.g., a weaker than normal or “sluggish” pupil response) (33).

### Statistical Analysis

The results are expressed as mean ± standard deviation, median, 1st quartile (q1), 3rd quartile (q3), interquartile range, count, and percentage frequency. No sample size calculation was performed due to the retrospective design of this study. Shapiro–Wilk test was used to assess the normal distribution of continuous variables. The null hypothesis of the Shapiro-Wilk test is that the population is normally distributed. For a *P* value less than the conventional alpha level (alpha = 0.05), the null hypothesis is rejected, and the data tested are assumed as not normally distributed. In this case, a non-parametric test for comparison should be used. Mann–Whitney *U*-test was used to compare continuous variables, while categorical variables were compared with Fisher's exact test. Patient survival was evaluated by using the Kaplan–Meier estimator. Log-rank test was used to compare the survival curves. Continuous and categorical variables were entered into univariate Cox proportional hazard regression models. Efron approximation was used for each Cox model. The proportional hazards assumption for each significant Cox regression model was evaluated using correlation coefficients between transformed survival times and scaled Schoenfeld residuals. Significant variables to univariate Cox regression were entered in the multivariate model, with regression coefficient and hazard ratio (HR) with the 95% confidence interval (CI) as the main outputs. A forest plot and a rank-hazard plot were provided for multivariate Cox regression. The rank-hazard plot is able to visualize the relationship between the relative hazard of variables entered in the multivariate Cox regression model (34). Logistic regression was performed to assess the risk factors associated with neurological complications. The Hosmer–Lemeshow omnibus test was used for goodness-of-fit evaluation of each significant logistic regression model. Only logistic regression models that passed the goodness-of-fit test (*P* > 0.05) were presented. Significant variables to univariate logistic regression were entered in the multivariate model, with regression coefficient and odds ratio with the 95% confidence interval as the main outputs. A receiver operating characteristic curve was calculated for the multivariate logistic regression model as well as sensibility and specificity. Statistical significance was assumed in each test directly related to the study outcomes with a two-tailed *P*-value < 0.05. Statistical analysis was carried out by using the R software/environment (version 3.6.3, R Foundation for Statistical Computing, Vienna, Austria).

## Results

During the study period, 116 patients with COVID-19 were admitted to the SMPH ICU. Twenty-two patients were excluded because they did not meet the inclusion criteria. Thus, 94 patients were included in the final analysis, of whom 53 underwent noninvasive neuromonitoring. Thirty patients (56.6%) underwent repeated measures on different days during their ICU stay period. The whole repeated measurements ranged from 2 to 10 (4.86 ± 2.22 measurements), while the first and the last measurements were performed between the 1st and 33rd ICU day (mean delta: 14.8 ± 9.22 days).

### Overall Population

**Figure 1:**
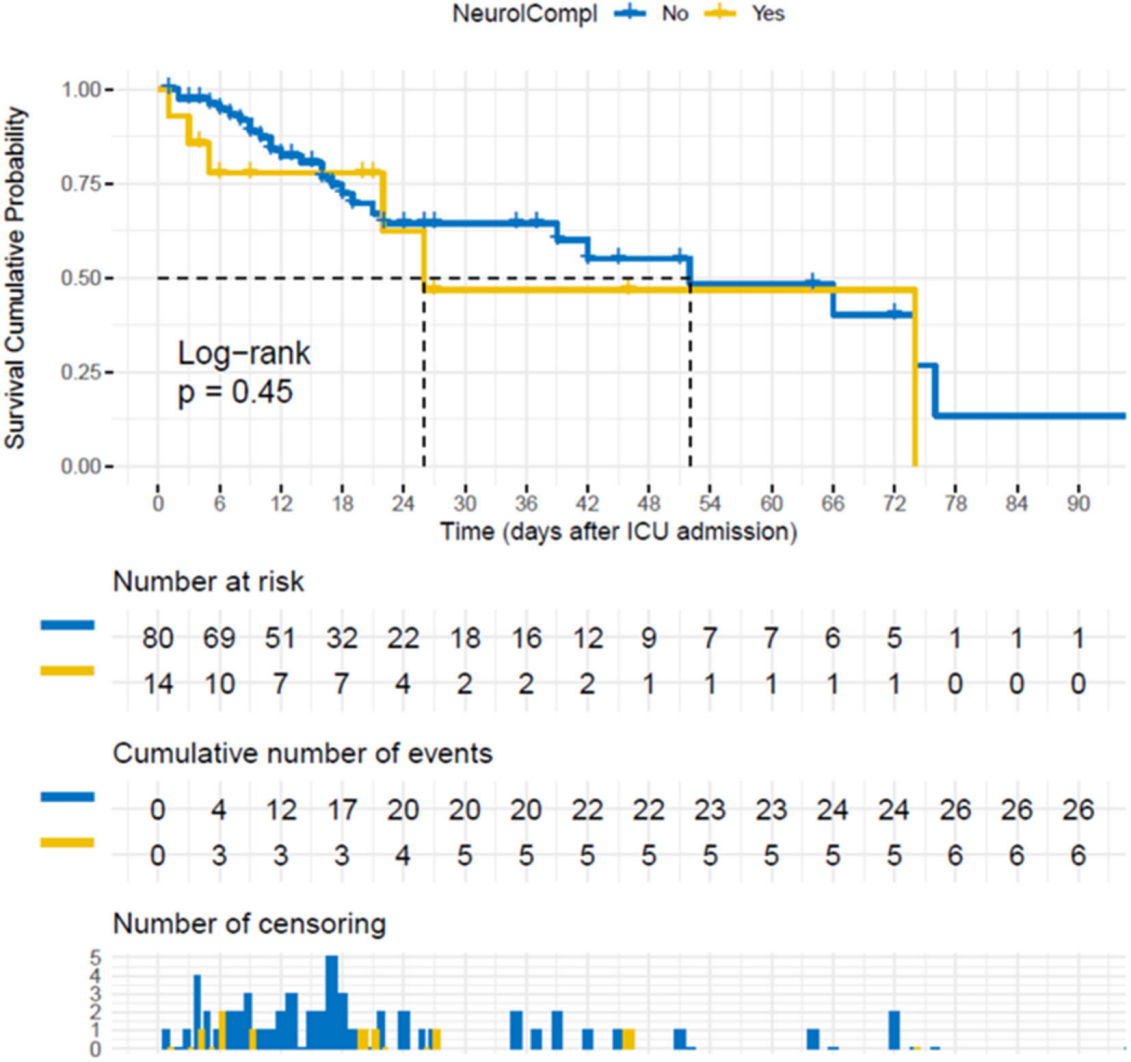
Survival cumulative probability after intensive care unit (ICU) admission for the 94 patients included. Survival cumulative probability after ICU admission for the patients (*n* = 94) who fulfilled the inclusion criteria, stratifying for the absence/presence (no/yes) of neurological complications.

The characteristics of the 94 patients admitted to our ICU who fulfilled the inclusion criteria—with and without neurological complications—are described in Table 1. Neurological complications were detected in 47/94 patients (50%). Nine patients presented more than one neurological complication. The most common complications are reported in Table 2. The occurrence of neurological complications did not result in increased ICU mortality (*p* = 0.450) (Figure 1) but was associated with longer hospital (36.77 ± 25.14 vs. 19.43 ± 16.86 days, *p* < 0.001) and ICU (31.51 ± 22.64 vs. 11.51±10.14; *p* < 0.001) stay compared to the absence of neurological complications (Table 1).

**Table 1:** Demographic and clinical characteristics of the COVID-19 patients included in the study.

**Characteristics**	**All patients** **(*n* = 94)**	**Patients with neurological complications** **(*n* = 47)**	**Patients without neurological complications** **(*n* = 47)**
Gender [male, *n* (%)]	74 (78.7%)	41 (87.2)	33 (70.2)
Age (y/o, mean ± SD)	61.6 ± 11.1	62.4 ± 8.3	60.8 ± 13.3
Weight (kg, mean ± SD)	90.0 ± 13.6	82.9 ± 14.2	80.5 ± 13.0
Height (cm, mean ± SD)	176.0 ± 7.9	171.7 ± 7.5	171.8 ± 8.3
BMI (kg/m^2^, mean ± SD)	29.9 ± 4.2	28.1 ± 4.2	27.3 ± 4.1
**Comorbidities [*****n*****, (%)]**			
Hypertension	49 (52.1)	27 (57.4)	22 (46.8)
Chronic renal disease	5 (5.3)	5 (10.6)	0 (0.0)
Diabetes	14 (14. 9)	6 (12.8)	8 (17.0)
Chronic respiratory disease	0 (0.0)	0 (0.0)	0 (0.0)
Chronic liver disease	3 (3.2)	1 (2.1)	2 (4.3)
Cancer	6 (6.4)	3 (6.4)	3 (6.4)
Cardiac failure	8 (8.5)	5 (10.6)	3 (6.4)
Neurological disease	0 (0.0)	0 (0.0)	0 (0.0)
Hospital length of stay (days, mean ± SD)	28.10 ± 23.00	36.77 ± 25.14	19.43 ± 16.86
ICU length of stay (days, mean ± SD)	21.51 ± 20.14	31.51 ± 22.64	11.51 ± 10.14
**ICU outcome [*****n*****, (%)]**			
Alive	61 (64.90)	31 (65.95)	30 (63.83)
Critical	2 (2.10)	2 (4.25)	0 (0.00)
Death	31 (33)	14 (29.78)	17 (36.17)
Days of mechanical ventilation (days, mean ± SD)	20.00 ± 16.33	22.93 ± 19.62	8.85 ± 7.75
Days from symptoms to hospital admission (days, mean ± SD)	3.98 ± 10.11	3.81 ± 7.16	4.14 ± 12.42
Days from symptoms to ICU admission (days, mean ± SD)	10.92 ± 6.84	9.51 ± 6.73	12.30 ± 6.74
Higher D-dimer during ICU stay (ng/ml, mean ± SD)	17.636 ± 26.631	14.067 ± 21.401	17.878 ± 31.310
Higher CRP during ICU stay (mg/L, mean ± SD)	266.25 ± 120.88	232.78 ± 127.49	161.47 ± 102.81
Lower PaO_2_ during ICU stay (mmHg, mean ± SD)	60 ± 10.92	52.97 ± 7.80	57.96 ± 13.03

*n*, number; SD, standard deviation; y/o, years old; BMI, body mass index; ICU, intensive care unit; PaO_2_, partial pressure of oxygen; CRP, C-reactive protein.

**Table 2:** Type and incidence of neurological complications in the overall intensive care unit population.

**Neurological complications**	**Number of patients** **(%)**
Overall	47 (50)
Delirium	34 (36.17)
Critical illness neuropathy	5 (5.32)
Coma	4 (4.25)
Acute ischemic stroke	3 (3.19)
Stupor	3 (3.19)
Seizures	2 (2.13)
Encephalopathy	2 (2.13)
Cognitive deficit	1 (1.06)
Depression	1 (1.06)

### Risk of Developing Neurological Complications

**Figure 2:**
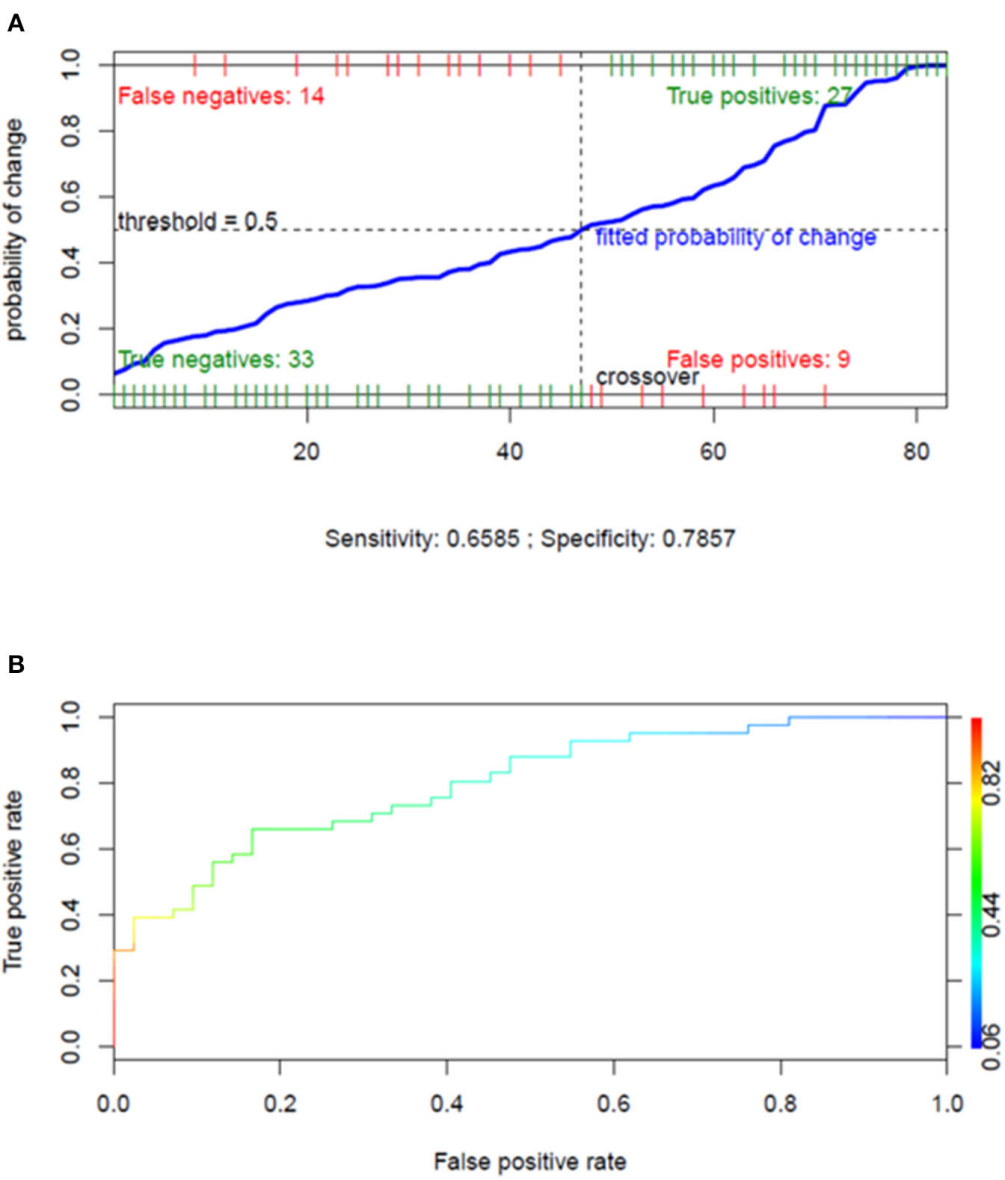
Performance of the multivariate logistic regression model for assessing the factors independently associated with the risk of neurological complications. **(A)** Overall performance of the multivariate logistic regression model presented in Table 3 (dependent variable: neurological complications; independent variables: days of mechanical ventilation and C-reactive protein). **(B)** Receiver operating characteristic curve of the same multivariate logistic regression model (area under the curve = 0.818).

On univariate logistic regression, duration of mechanical ventilation and CRP values were associated with the risk of developing neurological complications (Table 3). Multivariate logistic regression demonstrated that the duration of mechanical ventilation was independently associated with the risk of neurological complications (OR: 1.1; 95% CI: 1.046–1.175; *p* = 0.001) (Table 3), with an area under the curve of 0.818, sensitivity of 0.658, and specificity of 0.786 (Figure 2). Additional results concerning the cumulative survival probability of the overall population after hospital and ICU admission are shown in Supplementary Figures 1–3.

**Table 3:** The significant variables associated with neurological complications as assessed by univariate logistic regression and the output of the subsequent multivariate model, for the patients (*n* = 94) who fulfilled the inclusion criteria.

**Variable**	**Univariate**				**Multivariate**			
	**RC**	**OR**	**95% CI**	***P*-value**	**RC**	**OR**	**95% CI**	***P*-value**
Days of mechanical ventilation	0.088	1.092	1.046–1.154	<0.001	0.095	1.100	1.046–1.175	0.001
CRP	0.005	1.005	1.002–1.009	0.006	0.002	1.002	0.997–1.006	0.443

*CRP, C-reactive protein; RC, regression coefficient; OR, odds ratio; CI, confidence interval*.

### Noninvasive Neuromonitoring Population

A total of 53 patients underwent noninvasive neuromonitoring. The characteristics of this subgroup are described in Supplementary Table 5. TCD was performed in 51/53 (96.23%), ONSD in 49/53 (92.45%), and automated pupillometry in 29/53 (54.72%) patients. The median sFV was 99.50 (q1: 87.00; q3: 108.75) cm/s, the median dFV was 31.59 (q1: 22.87; q3: 45.00) cm/s, and the median PI was 1.16 (q1: 0.99; q3: 1.41). The median ONSD was 5.65 (q1: 4.80; q3: 6.60) mm. The median nICP_TCD_ was 17.57 (q1: 12.68; q3: 25.21) mmHg, and the median nICP_ONSD_ was 14.33 (q1: 10.07; q3: 19.33) mmHg.

### Effect of Altered Neuromonitoring Findings on Patients' Outcome

**Figure 3:**
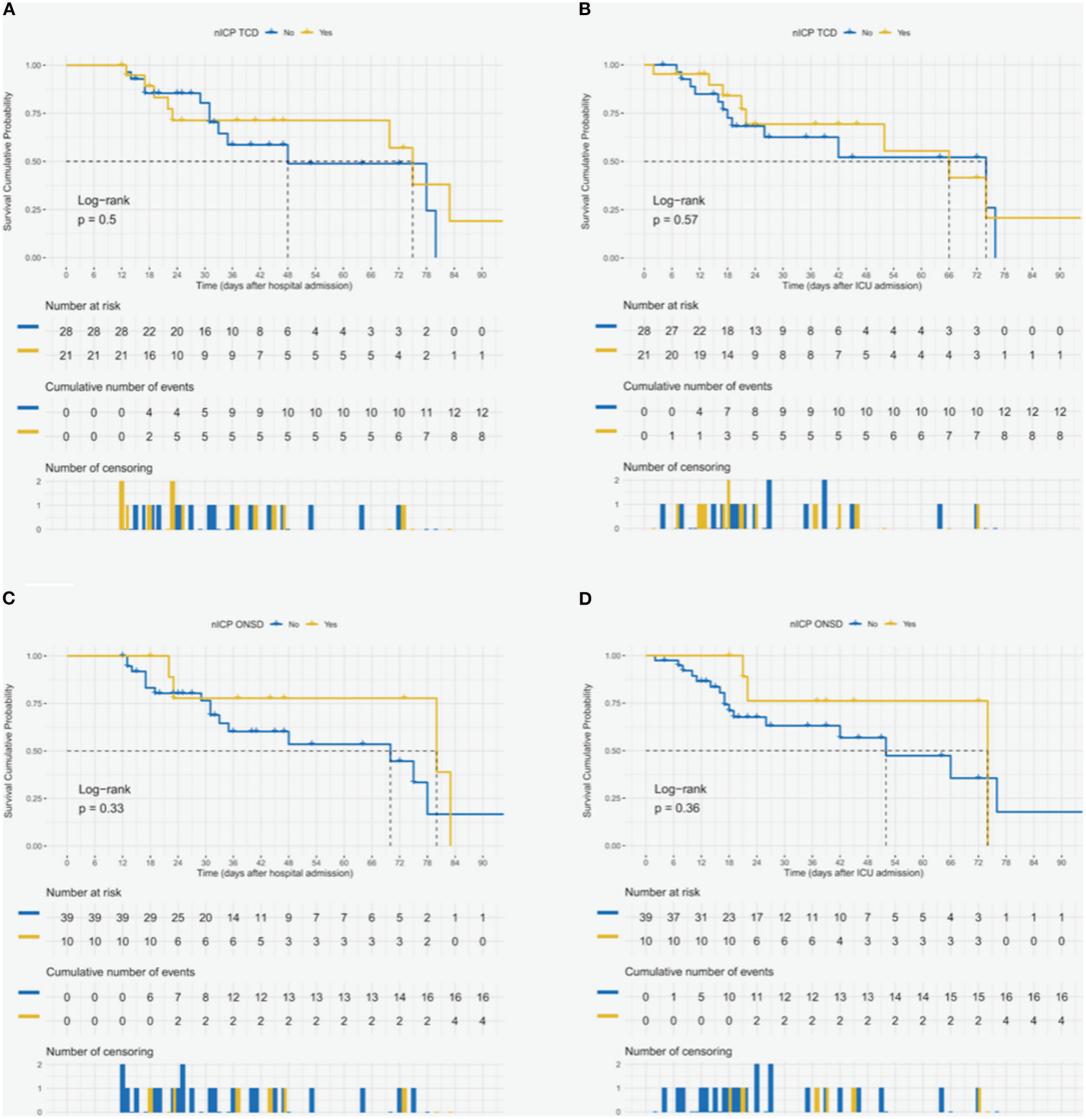
Survival cumulative probability after hospital and intensive care unit (ICU) admission for the patients who underwent noninvasive neuromonitoring. Survival cumulative probability after hospital and ICU admission for the patients (*n* = 49) who underwent noninvasive intracranial pressure, monitoring with both transcranial Doppler **(A,B)** and optic nerve sheath diameter **(C,D)**.

High ICP was found in 21 nICP_TCD_ patients (39.62%) and in 10 nICP_ONSD_ patients (18.87%). Among the 29 patients who underwent automated pupillometry, nine (31.03%) presented altered pupillary reactivity. Patients with increased nICP_ONSD_ and nICP_TCD_, compared to those with normal nICP_ONSD_ and nICP_TCD_, did not experience a longer hospital stay (nICP_ONSD_: 45.00 ± 25.27 vs. 36.33 ± 24.70 days, *p* = 0.222; nICP_TCD_: 38.90 ± 30.34 vs. 35.43 ± 19.23 days, *p* = 0.691), but patients with higher nICP_ONSD_ had longer ICU stays (nICP_ONSD_: 42.30 ± 23.21 vs. 28.26 ± 22.28 days, *p* = 0.042; nICP_TCD_: 32.86 ± 25.55 vs. 28.61 ± 20.89 days, *p* = 0.721). Additional descriptive data on TCD are reported in Supplementary Table 6. Patients with increased ICP according to ONSD and TCD values compared to those with normal ICP showed no differences in hospital or ICU mortality (Figure 3) (Supplementary Table 7). The outcomes of the Cox regression models for the patients who underwent noninvasive neuromonitoring are reported in Supplementary Table 8 and Supplementary Figures 4, 5. The significant variables associated with neurological complications assessed by univariate logistic regression and the output of the subsequent multivariate model, for the patients (*n* = 53) who underwent noninvasive neuromonitoring, are reported in Table 4. A brief case report describing the serial measurements and the course of the disease is presented in Supplementary Case 1.

**Table 4:** The significant variables associated with neurological complications as assessed by univariate logistic regression and the output of the subsequent multivariate model, for the patients (*n* = 53) who underwent noninvasive neuromonitoring.

**Variable**	**Univariate**			**Multivariate**				
	**RC**	**OR**	**95% CI**	***P*-value**	**RC**	**OR**	**95% CI**	***P*-value**
Days between hospital and ICU admission	−0.092	0.912	0.815–0.988	0.058	−0.082	0.921	0.815–1.003	0.114
dFV	−0.049	0.952	0.906–0.994	0.036	−0.044	0.956	0.909–1.001	0.069

*ICU, intensive care unit; dFV, diastolic flow velocity; RC, regression coefficient; OR, odds ratio; CI, confidence interval*.

## Discussion

The main findings of our study are as follows: (1) neurological complications are common in COVID-19 patients and have no effect on mortality but can be associated with increased hospital and ICU length of stay, (2) the duration of mechanical ventilation is independently associated with the development of neurological complications, and (3) increased ICP (estimated by ONSD) and pupillary abnormalities are common and associated with longer ICU length of stay.

To our knowledge, this is the first study describing cerebrovascular dynamics in mechanically ventilated COVID-19 patients, which could potentially help to elucidate the underlying pathophysiology of the neurological complications in this patient population. Moreover, to date, no studies have taken into account the possible secondary effects of mechanical ventilation and inflammation on neurological outcome.

There are several theories concerning the central and the peripheral neurological changes following a SARS-CoV-2 infection: viral neurotropism, including trans-synaptic spread, endothelial or lymphocyte invasion by SARS-CoV-2, a hyperinflammatory and hypercoagulative state, or even mechanical ventilation-associated impairment (35). In our cohort, neurological complications were detected in half of the patients admitted to our ICU with confirmed COVID-19 pneumonia who fulfilled the inclusion criteria. The most frequent complication was delirium (36.70%), followed by coma, critical illness neuropathy, ischemic stroke, stupor, encephalopathy, seizures, cognitive deficit, and depression. The frequency of delirium is in line with current COVID-19 literature, in which it has ranged from 26.80 to 73.60% (34, 36). Delirium was identified both in the acute and in the post-ICU phases during the severe acute respiratory syndrome (SARS) and Middle-East respiratory syndrome (MERS) epidemics, with a possible detrimental effect on length of stay (37). Sedatives, analgesics, pain, psychological stressors, hypoxia, metabolic and electrolyte imbalances, infection, hyperthermia, sepsis, mechanical ventilation, light, and the use of physical restraints are well-known contributors to delirium occurrence in the ICU (38, 39). Delirium is known to be associated with longer ICU stay and mechanical ventilation days as well as an increased risk of death at 6 months, disability, and long-term cognitive dysfunction (39, 40). Our results are in line with these findings; patients who developed neurological complications (mainly delirium) did not show increased ICU mortality, but they did have prolonged hospital and ICU stays, often exceeding 2 weeks, with a major impact on health expenditures and resource utilization—especially in the resource-limited setting of a pandemic.

Mechanical ventilation days and inflammation (assessed by C-reactive protein) were associated with the occurrence of neurological complications at the univariate analysis. This suggests that the magnitude of the inflammatory response and the severity of respiratory impairment may strongly affect the occurrence of neurological complications in COVID-19 (35).

Several cerebral hemodynamic changes occurred in the subpopulation undergoing neuromonitoring. First, patients with COVID-19 presented higher median ONSD values compared to the normal population [5.65 mm (4.80–6.60) vs. 4.10 mm (3.85–4.35) (41)]. As described in the literature, the threshold of increased nICP_ONSD_ is 5–6 mm (27); this suggests that increased ICP is a common finding in COVID-19 patients. In fact, increased ICP measured with both ONSD and TCD was very common, and a large portion of patients (38.71%) exhibited altered pupillary reactivity.

Several factors can potentially cause increased intracranial pressure in patients with respiratory failure and pneumonia, including increased PaCO_2_, which can cause cerebral vasodilatation (42, 43), or the use of high PEEP and consequently increased intrathoracic pressure (44). Indeed we found that PEEP was higher in those who showed higher nICP, whether assessed by ONSD or TCD (as we reported in the Supplementary Material). Although the difference was not statistically significant, it suggests that mechanical ventilation can interfere widely with cerebral hemodynamics.

Although common, the occurrence of increased ICP had no effect on cumulative probability of survival; it did prolong ICU-LOS when measured by ONSD, but not by TCD. This confirms that, in COVID-19 patients, noninvasive ICP monitoring may be essential for the early detection of patients who are at risk of longer ICU-LOS with subsequent complications and difficult recovery. The incongruity between the results of the two noninvasive methods might be explained by differences in pathophysiological sensitivity and specificity for ICP assessment between the two (26); both techniques can present important methodological limitations (intra-interobserver variability, artifacts, and low accuracy in estimating ICP as a number) (28). We therefore recommend a multimodal monitoring approach for the noninvasive measurement of intracranial pressure to predict neurological complications (28). Although we found no correlation between altered neuromonitoring findings and the occurrence of neurological complications, we strongly recommend the use of these methods in critically ill patients with COVID-19 and, in general, in ICU patients undergoing mechanical ventilation for the early detection of neurological complications. Noninvasive neuromonitoring tools are safe, quick, low-cost, and easily available and can provide relevant data at the patients' bedside.

## Limitations

This study has several limitations which must be addressed. First, this was a retrospective study of prospectively collected data. Data were collected within the clinical context of the COVID-19 pandemic (limited availability of personal protective equipment, clinical reasons, and so on). Thus, neuromonitoring data are neither complete nor available for all patients. Second, TCD, ONSD, and automated pupillometer measurements were intermittent and were obtained at different stages of the patients' ICU stays. Continuous, daily, standardized monitoring would have provided more accurate data on the behavior of cerebrovascular hemodynamics in this population. Because of the critical demands of the pandemic, we were unable to obtain multiple neuromonitoring measurements to reduce intra- and inter-observer variability among the operators. However, our team consists of a group of specialized physicians with ample experience in the use of noninvasive monitoring. Third, we did not use other methods—such as neuroimaging or lumbar puncture—to confirm the findings of intracranial hypertension. Fourth, the relatively small sample size of our study, which depended on the number of COVID-19 patients admitted to our ICU and was thus beyond our control, limits the strength of our conclusions and results. Fourth, since this is not an interventional study, the sedation and analgesia protocols were not standardized but rather were based on the clinical needs of the patients, which may have had an impact on FV, ONSD, and automated pupillometer-derived values. Fifth, in this study population, ICP was only moderately elevated due to factors not related to intracranial pathologies, which might explain why the neurological complications did not lead to life-threatening complications.

## Conclusions

Neurological complications, particularly delirium, are common in COVID-19 patients and are associated with longer hospital and ICU stay. The duration of mechanical ventilation is strongly associated with the development of neurological complications. Noninvasive neuromonitoring during ICU stay may be helpful to detect cerebrovascular alterations earlier. Further studies, including a larger number of patients, may provide new insights on the role of noninvasive neuromonitoring in non-COVID-19 patients admitted to ICU for different pathologies.

## Data Availability Statement

The raw data supporting the conclusions of this article will be made available by the authors, without undue reservation.

## Ethics Statement

The studies involving human participants were reviewed and approved by the Ethics Committee of Liguria, Italy (registry number 163/2020). Written informed consent for participation was not required for this study in accordance with the national legislation and the institutional requirements.

## Author Contributions

DB and CR conceived the study, designed the study, acquired data, interpreted the data, and drafted the manuscript. GS analyzed and interpreted the data, critically revised the manuscript, and gave final approval. KC, FI, and MBL contributed to the acquisition of data, critical revision of the manuscript, and final approval. FT, LB, DG, AV, MBT, PR, NP, and IB contributed to the critical revision of the manuscript and final approval. PP helped with the study design, interpretation of the data, critical revision of the manuscript, and final approval. All authors contributed to the article and approved the submitted version.

## GECOVID-19 Collaborators

Chiara Berri, Anesthesia and Intensive Care, San Martino Policlinico Hospital, IRCCS for Oncology and Neurosciences, Genoa, Italy; Serena Cavalcoli, Anesthesia and Intensive Care, San Martino Policlinico Hospital, IRCCS for Oncology and Neurosciences, Genoa, Italy, Department of Surgical Sciences and Integrated Diagnostic (DISC), University of Genoa, Genoa, Italy; Elena Ciaravolo, Anesthesia and Intensive Care, San Martino Policlinico Hospital, IRCCS for Oncology and Neurosciences, Genoa, Italy, Department of Surgical Sciences and Integrated Diagnostic (DISC), University of Genoa, Genoa, Italy; Marcus Ferretti, Anesthesia and Intensive Care, San Martino Policlinico Hospital, IRCCS for Oncology and Neurosciences, Genoa, Italy; Maurizio Loconte, Anesthesia and Intensive Care, San Martino Policlinico Hospital, IRCCS for Oncology and Neurosciences, Genoa, Italy; Marco Sottano, Anesthesia and Intensive Care, San Martino Policlinico Hospital, IRCCS for Oncology and Neurosciences, Genoa, Italy, Department of Surgical Sciences and Integrated Diagnostic (DISC), University of Genoa, Genoa, Italy; Stefano Nogas, Anesthesia and Intensive Care, San Martino Policlinico Hospital, IRCCS for Oncology and Neurosciences, Genoa, Italy, Department of Surgical Sciences and Integrated Diagnostic (DISC), University of Genoa, Genoa, Italy; Paolo Frisoni, Anesthesia and Intensive Care, San Martino Policlinico Hospital, IRCCS for Oncology and Neurosciences, Genoa, Italy; Pasquale Anania, Department of Neurology, Rehabilitation, Ophthalmology, Genetics, Maternal and Child Health, (DINOGMI), University of Genoa, Genoa, Italy; Pietro Fiaschi, Department of Neurology, Rehabilitation, Ophthalmology, Genetics, Maternal and Child Health, (DINOGMI), University of Genoa, Genoa, Italy, Department of Neurosurgery, San Martino Policlinico Hospital, IRCCS for Oncology and Neurosciences, Genoa, Italy; Alessandro Prior Department of Neurology, Rehabilitation, Ophthalmology, Genetics, Maternal and Child Health, (DINOGMI), University of Genoa, Genoa, Italy; Alessandro Bertuccio, Neurosurgical Unit, Surgical Department, Azienda Ospedaliera SS. Antonio e Biagio e Cesare Arrigo, Alessandria, Italy; Angelo Schenone, Department of Neurosurgery, San Martino Policlinico Hospital, IRCCS for Oncology and Neurosciences, Genoa, Italy; Gianluigi Zona, Department of Neurology, Rehabilitation, Ophthalmology, Genetics, Maternal and Child Health, (DINOGMI), University of Genoa, Italy, Department of Neurosurgery, San Martino Policlinico Hospital, IRCCS for Oncology and Neurosciences, Genoa, Italy; Angelo Gratarola, Anesthesia and Intensive Care, San Martino Policlinico Hospital, IRCCS for Oncology and Neurosciences, Genoa, Italy; Flavio Villani, Division of Clinical Neurophysiology and Epilepsy Center, San Martino Policlinico Hospital, IRCCS for Oncology and Neurosciences, Genoa, Italy.

## Conflict of Interest

The authors declare that the research was conducted in the absence of any commercial or financial relationships that could be construed as a potential conflict of interest.

## Abbreviations

CI: confidence interval
COVID-19: coronavirus disease 2019
CPP: cerebral perfusion pressure
dFV: diastolic flow velocity
ESM: electronic supplemental material
FiO_2_: fraction of inspired oxygen
ICP: intracranial pressure
ICU: intensive care unit
IQR: interquartile range
LOS: length of stay
MAP: mean arterial pressure
MCA: middle cerebral artery
MERS: Middle East respiratory syndrome
mFV: mean flow velocity
nICP: noninvasive intracranial pressure
nICP_ONSD_: noninvasive intracranial pressure measured by optic nerve sheath diameter
nICP_TCD_: noninvasive intracranial pressure measured by transcranial Doppler
ONSD: optic nerve sheath diameter
OR: odds ratio
PaCO_2_: partial pressure of carbon dioxide
PaO_2_: partial pressure of oxygen
PcPs: pressure control or pressure support
PEEP: positive end-expiratory pressure
PI: pulsatility index
q1: 1st quartile
q3: 3rd quartile
ROC: receiver operating characteristic
RT-PCR: reverse transcriptase-polymerase chain reaction
SARS-CoV-2: severe acute respiratory syndrome coronavirus-2
SARS: severe acute respiratory syndrome
sFV: systolic flow velocity
SMPH: San Martino Policlinico Hospital
TCD: transcranial Doppler.
