# Supplementary material for: Neurological Complications and Noninvasive Multimodal Neuromonitoring in Critically Ill Mechanically Ventilated COVID-19 Patients
Source: Front Neurol. 2020 Nov 27;11:602114. doi: 10.3389/fneur.2020.602114 (PMC7729072; doi:10.3389/fneur.2020.602114)

**Electronic Supplemental Material (ESM)**

**Neurological complications and non-invasive multimodal neuromonitoring in critically ill COVID-19 patients**

Denise Battaglini^1,2^, Gregorio Santori^3^, Karthikka Chandraptham^1,3^, Francesca Iannuzzi^1,3^, Matilde Bastianello^1,3^, Fabio Tarantino^1^, Lorenzo Ball^1,3^, Daniele Roberto Giacobbe^4^, Antonio Vena^4^, Matteo Bassetti^4,5^, Matilde Inglese^6^, Antonio Uccelli^6^, Patricia Rieken Macedo Rocco^7^, Nicolò Patroniti^1,3^, Iole Brunetti^1^, Paolo Pelosi^1,3^, Chiara Robba^1^

for the *GECOVID-19 Group

^1^ Anesthesia and Intensive Care, San Martino Policlinico Hospital, IRCCS for Oncology and Neurosciences, Genoa, Italy.

^2^ Department of Medicine (DIMI), University of Barcelona (UB), Barcelona, Spain.

^3^ Department of Surgical Sciences and Integrated Diagnostic (DISC), University of Genoa, Genoa, Italy.

^4^ Department of Infectious Diseases, San Martino Policlinico Hospital - IRCCS for Oncology and Neurosciences, Genoa, Italy.

^5^ Department of Health Sciences (DISSAL), University of Genoa, Genoa, Italy.

^6^ Department of Neurology, Rehabilitation, Ophthalmology, Genetics, Maternal and Child Health, (DINOGMI), University of Genoa, Italy.

^7^ Laboratory of Pulmonary Investigation, Carlos Chagas Filho Institute of Biophysics, Federal University of Rio de Janeiro, Rio de Janeiro, RJ, Brazil.

**Tables of Contents**

**Definition of neurological complications** [p. 2].

**ESM - Table 1.** Richmond Agitation Sedation Scale (RASS) [p. 4].

**ESM - Table 2.** Glasgow Outcome Scale (GOS) [p. 4].

**ESM - Table 3.** Modified Rankin scale [p. 4].

**ESM - Table 4**. Confusion Assessment Method for the ICU (CAM-ICU) [p. 5].

**ESM - Figure 1**. The survival cumulative probability of the patients (n = 94) who fulfilled the inclusion criteria after hospital admission [p. 6].

**ESM - Figure 2**. The survival cumulative probability of the patients (n = 94) who fulfilled the inclusion criteria after ICU admission [p. 7].

**ESM - Table 5.** Characteristics of the patients who underwent noninvasive neuromonitoring [p. 8]

**Transcranial Doppler – descriptive data** [p. 9].

**ESM - Table 6**. TCD on the right and left mean cerebral arteries [p. 9].

**Sedation analgesia** [p. 9].

**ESM - Figure 3**. Survival cumulative probability at 90 days after hospital and ICU admission in the whole population of COVID-19 patients (n = 116), stratifying for not receiving / receiving (No/Yes) non-invasive neuromonitoring [p. 10].

**ESM - Table 7.** Comparison between normal and high intracranial pressure, calculated by transcranial Doppler and optic nerve sheath diameter [p. 11].

**Cox regression models in the the patients who underwent non-invasive neuromonitoring** [p. 12].

**ESM - Table 8.** The significant variables associated with survival assessed by univariate Cox regression and the output of the subsequent multivariate model, for the patients (n = 53) who underwent non-invasive neuromonitoring [p. 12].

**ESM - Figure 4.** Forest plot of the variables entered in the multivariate Cox regression model, for the patients (n = 53) who underwent non-invasive neuromonitoring [p. 13].

**ESM - Figure 5.** Rank-hazard plot of the variables entered in the multivariate Cox regression model, for the patients (n = 53) who underwent non-invasive neuromonitoring [p. 14].

**Definition of neurological complications**

Ischemic stroke: neurological deficit caused acute focal injury for vascular involvement(1). Intracranial hemorrhage: bleeding inside the skull. Hemorrhagic stroke: neurological deficit caused by an acute focal injury for vascular involvement with intracerebral or subarachnoid hemorrhage(1). Subdural hematoma: blood collection under the dura mater(2). Encephalitis/meningitis: severe inflammatory disorder of the brain or meninges(3). Coma: state of deep unconsciousness, with closed eyes and unresponsive state(4). Transverse myelitis and other spinal cord pathology: inflammatory disorder with acute or subacute motor-sensory and autonomic spinal cord dysfunction(5). Seizures: disease of the brain with at least two unprovoked seizures in >24 hours; one unprovoked seizure and a probability of further seizures similar to the general recurrence risk (at least 60%) after two episodes of seizures within 10 years; clear diagnosis of epilepsy syndrome(6) and non-convulsive epileptic status(7). Delirium: acute change in consciousness and attention caused by an organic condition, evaluated by the CAM-ICU assessment(8). Guillain-Barré Syndrome or variants: acute onset of inflammatory immune-mediated polyradiculoneuropathy that presents with progressive weakness, tingling, autonomic disfunction and pain(5). Critical illness myopathy/neuropathy: neuromuscular weakness acquired in the intensive care setting(9). Hypogeusia/hyposmia: quantitative taste and smell disorders(9). Delirium: hyperactive, hypoactive, or mixed label status depending on the level of arousal. Stupor: unresponsive state that can be modified by repeated stimuli(10). Cognitive deficits: inability to learn, solve problems, remember, and access to stored informations(11). Depression: mood disorder that cause feeling of sadness and loss of interest(12).

**References**

1. Sacco RL, Kasner SE, Broderick JP, Caplan LR, Connors JJ, Culebras A, Elkind MSV, George MG, Hamdan AD, Higashida RT, et al. An updated definition of stroke for the 21st century: A statement for healthcare professionals from the American heart association/American stroke association. *Stroke* (2013) **44**:2064–2089.

2. Stone JL, Rifai MH, Sugar O, Lang RG, Oldershaw JB, Moody RA. Subdural hematomas. I. Acute subdural hematoma: progress in definition, clinical pathology, and therapy. *Surg Neurol* (1983) **19**:216–31.

3. Graus F, Titulaer MJ, Balu R, Benseler S, Bien CG, Cellucci T, Cortese I, Dale RC, Gelfand JM, Geschwind M, et al. A clinical approach to diagnosis of autoimmune encephalitis. *Lancet Neurol* (2016) **15**:391–404.

4. Kadapatti K, Iyer SK. “Coma,” in *ICU Protocols: A Step-wise Approach, Vol I*, 319–325. doi:10.1007/978-981-15-0898-1_32

5. Rodríguez Y, Rojas M, Pacheco Y, Acosta-Ampudia Y, Ramírez-Santana C, Monsalve DM, Gershwin ME, Anaya JM. Guillain–Barré syndrome, transverse myelitis and infectious diseases. *Cell Mol Immunol* (2018) **15**:547–562.

6. Falco-Walter JJ, Scheffer IE, Fisher RS. The new definition and classification of seizures and epilepsy. *Epilepsy Res* (2018) **139**:73–79.

7. Holtkamp M, Meierkord H. Nonconvulsive status epilepticus: A diagnostic and therapeutic challenge in the intensive care setting. *Ther Adv Neurol Disord* (2011) **4**:169–181.

8. Setters B, Solberg LM. Delirium. *Prim Care* (2017) **44**:541–559.

9. Finsterer J, Stollberger C. Causes of hypogeusia/hyposmia in SARS-CoV2 infected patients. *J Med Virol* (2020)10.1002/jmv.25903. doi:10.1002/jmv.25903

10. Tindall SC. *Level of Consciousness*. 3rd editio. , ed. H. J. Walker HK, Hall WD Boston: Butterworths (2020). doi:10.32388/ajo2fj

11. Morley JE, Morris JC, Berg-Weger M, Borson S, Carpenter BD, del Campo N, Dubois B, Fargo K, Fitten LJ, Flaherty JH, et al. Brain Health: The Importance of Recognizing Cognitive Impairment: An IAGG Consensus Conference. *J Am Med Dir Assoc* (2015) **16**:731–739. doi:10.1016/j.jamda.2015.06.017

12. Chand SP, Arif H. *Depression*. Treasure Island (FL): Stat Pearls Publishing (2020). Available at: https://www.ncbi.nlm.nih.gov/books/NBK430847/ [Accessed July 20, 2020]

**Outcome scales**

**ESM - Table 1.** Richmond Agitation Sedation Scale (RASS).

| **Score** | **Term** | **Description** |
| --- | --- | --- |
| +4 | Combative | Overtly combative or violent; immediate danger to staff |
| +3 | Very agitated | Pulls on or removes tube(s) or catheter(s) or has aggressive behaviour toward staff |
| +2 | Agitated | Frequent non-purposeful movement or patient-ventilator desynchrony |
| +1 | Restless | Anxious or apprehensive but movements not aggressive or vigorous |
| 0 | Alert and calm | Spontaneously pays attention to caregiver |
| -1 | Drowsy | Not fully alert, but has sustained (more than 10 sec) awakening, with eye contact, to voice |
| -2 | Light sedation | Briefly (less than 10 sec) awakens with eye contact to voice |
| -3 | Moderate sedation | Any movement (but no eye contact) to voice |
| -4 | Deep sedation | No response to voice, but any movement to physical stimulation |
| -5 | Unresponsive | No response to voice or physical stimulation |

**ESM - Table 2.** Glasgow Outcome Scale (GOS).

| **Class** | **Definition** |
| --- | --- |
| 1 | Dead |
| 2 | Vegetative State |
| 3 | Severe Disability (able to follow commands, unable to live independently) |
| 4 | Moderate Disability (able to live independently, unable to return to work/school) |
| 5 | Good Recovery (able to return to work/school) |

**ESM - Table 3.** Modified Rankin scale.

| **Scale** | **Definition** |
| --- | --- |
| 0 | No symptoms |
| 1 | No significant disability. Able to carry out all usual activities with minimal symptoms. |
| 2 | Slight disability. Able to assess daily activity without assistance, unable to carry out all these activities. |
| 3 | Moderate disability. Requires assistance, unable to walk alone without help. |
| 4 | Moderately severe disability. Needs for assistance for own daily bodily needs, unable to walk alone without assistance. |
| 5 | Severe disability. Unable to attend own body needs without constant assistance, nursing care and attention. Incontinent. |
| 6 | Dead. |

**ESM - Table 4.** Confusion Assessment Method for the ICU (CAM-ICU).

| **CAM-ICU** | **Criteria** |
| --- | --- |
| 1. Alteration/Fluctuation in Mental Status | Is the patient’s mental status different than his/her baseline? OR Has the patient had a fluctuation in mental status in the past 24 hours as evidenced by fluctuation on a sedation scale (eg RASS, GCS)? |
| 1. Inattention: Alteration/Fluctuation in Mental Status | Tell the patient “I am going to read to you a series of 10 letters. Whenever you hear the letter A, squeeze my hand”. (Calculate errors >2) |
| 1. Altered Level of Consciousness (LOC) | Present if the RASS score is anything other than Alert and Calm (0) OR if SAS is anything other than calm (4) |
| 1. Disorganised thinking | Yes/no Questions:  Will a stone float on water?  Are there fish in the sea?  Does 1-pound weigh more than 2 pounds?  Can you use a hammer to pound a nail?  Commands: ask to follow your instructions:  Hold up these many fingers (hold 2 fingers in front of the patient)  Now do the same thing with the other hand (do not demonstrate the number of fingers this time) |
| If features 1 and 2 are both present and either Features 3 and 4 are present: CAM-ICU positive, delirium is present | |

**Supplemental Results**


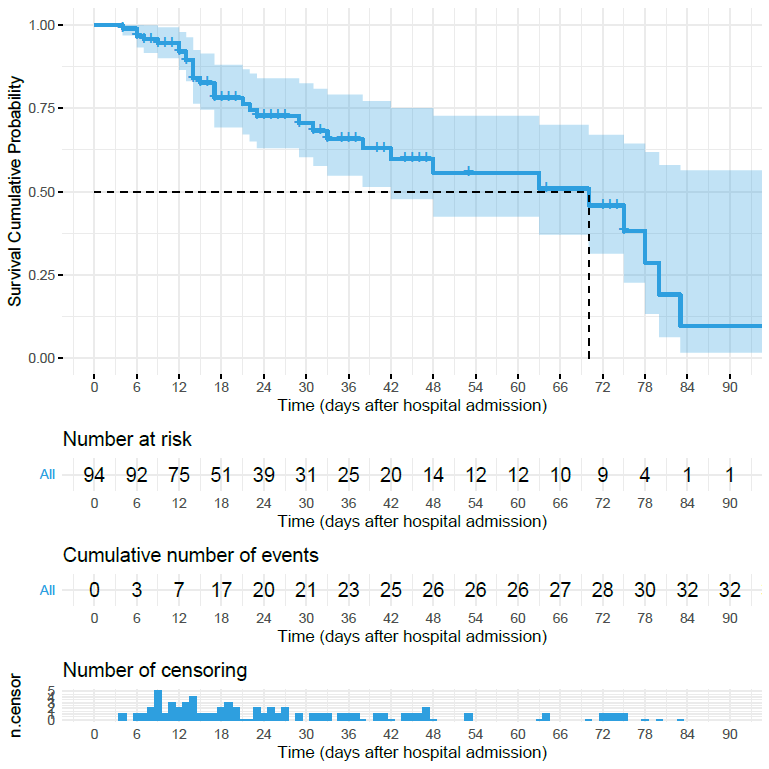


**ESM - Figure 1**. The survival cumulative probability of the patients (n = 94) who fulfilled the inclusion criteria after hospital admission [15 days (d): 0.826; 30 d: 0.705; 45 d: 0.598; 60 d: 0.555; 90 d: 0.095).


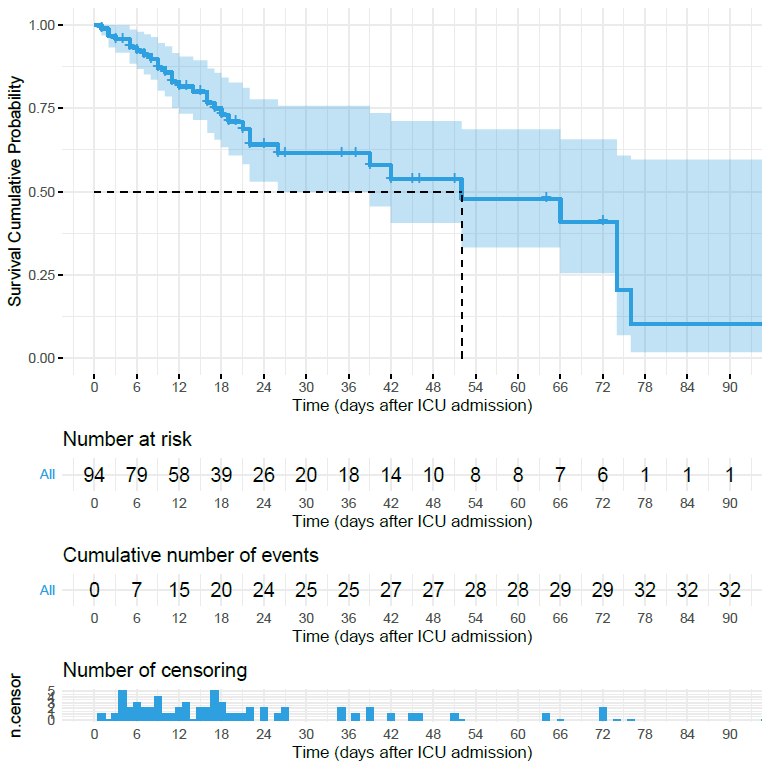


**ESM - Figure 2.** The survival cumulative probability of the patients (n = 94) who fulfilled the inclusion criteria after ICU admission [15 days (d): 0.799; 30 d: 0.615; 45 d: 0.537; 60 d: 0.478; 90 d: 0.102].

**Non-invasive neuromonitoring population**

**ESM - Table 5**. Demographic characteristics of the COVID-19 patients who underwent non-invasive neuromonitoring.

| **Characteristic** | **Patients who underwent**  **non-invasive neuromonitoring**  **(n = 53)** |
| --- | --- |
| Gender [male, n, (%)] | 41 (77.36) |
| Age (y/o, mean±SD) | 64.83 ± 7.87 |
| Weight (kg, mean±SD) | 81.03 ± 12.19 |
| Height (cm, mean±SD) | 171.89 ± 6.93 |
| BMI (kg/m^2^, mean±SD) | 27.44 ± 4.00 |
| Ventilation and gas exchange during neuromonitoring  Type [n, (%)]  Assisted  Controlled  Spontaneous breathing, COT  PEEP (cmH_2_O, mean±SD)  Plateau pressure (cmH_2_O, mean±SD)  Tidal volume (ml, mean±SD)  Respiratory rate (breaths/minutes, mean±SD)  FiO_2_ (mean±SD)  PaCO_2_ (mmHg, mean±SD)  PaO_2_ (mmHg, mean±SD) | 22 (41.51)  20 (37.74)  11 (20.75)  9.77 ± 2.79  23.47 ± 3.38  539.19 ± 119.30  20.19 ± 4.98  0.53 ± 0.19  48.59 ± 12.19  86.86 ± 22.33 |
| Comorbidities [n, (%)]  Hypertension  Chronic renal disease  Diabetes  Chronic respiratory disease  Chronic liver disease  Cancer  Cardiac failure  Neurological disease | 25 (47.17)  0 (0.00)  6 (11.32)  6 (11.32)  1 (1.89)  2 (3.77)  5 (9.43)  1 (1.89) |
| SOFA at ICU admission (days, mean±SD) | 4.42 ± 1.98 |
| GOS at ICU discharge (days, mean±SD) | 3.25 ± 0.51 |
| mRS at ICU discharge (days, mean±SD) | 3.28 ± 0.77 |
| Neurological complications [n, (%)]  Overall  Male  Female | 23 (43.40)  21 (91.30)  2 (8.69) |
| Status [n, (%)]  Alive  Critical  Death | 32 (60.38)  2 (3.77)  19 (35.85) |

n: number; SD: standard deviation; BMI: body mass index; ICU: intensive care unit; SOFA: sequential organ failure assessment; GOS: Glasgow outcome score; mRS: modified Rankin scale; COT: conventional oxygen therapy; PEEP: positive end-expiratory pressure; PaCO_2:_ partial pressure of carbon dioxide; PaO_2:_ partial pressure of oxygen.

**Transcranial Doppler – descriptive data**

Transcranial Doppler (TCD) was assessed on 51 patients. Descriptive statistic of TCD on the right and left mean cerebral artery (MCA) is reported in **ESM -** **Table 5**. Median intracranial pressure (ICP) assessed by TCD was calculated on 46 patients: 18.36 (q1: 10.73; q2: 37.51; IQR 26.78). Median ICP value including only patients who completed the follow-up (n = 44) was 17.64 (q1 = 10.43; q3 = 36.87; IQR = 26.44).

**ESM - Table 6.** TCD on the right and left mean cerebral arteries.

| **Side** | **TCD variables** | **Median** | **q_1_** | **q_3_** | **IQR** |
| --- | --- | --- | --- | --- | --- |
| Right MCA | sFV (cm/s) | 98.00 | 87.00 | 109.50 | 22.50 |
|  | dFV (cm/s) | 28.00 | 21.00 | 43.55 | 22.55 |
|  | PI | 1.24 | 0.87 | 1.65 | 0.78 |
|  | R | 0.68 | 0.55 | 0.79 | 0.24 |
|  | mFV (cm/s) | 51.53 | 44.67 | 62.06 | 17.39 |
| Left MCA | sFV (cm/s) | 98.00 | 85.22 | 106.50 | 21.28 |
|  | dFV (cm/s) | 32.00 | 20.50 | 45.70 | 25.20 |
|  | PI | 1.13 | 0.84 | 1.67 | 0.82 |
|  | R | 0.65 | 0.54 | 0.80 | 0.25 |
|  | mFV (cm/s) | 50.35 | 43.88 | 63.92 | 20.03 |

TCD, transcranial doppler; q1, first quartile; q3, third quartile; IQR, interquartile range; MCA, mean cerebral artery; sFV, systolic flow velocity; dFV, diastolic flow velocity; PI, pulsatility index; R, resistances; mFV, mean flow velocity.

**ESM - Sedation-analgesia**

During neuromonitoring, 16 patients (30.19%) were deeply sedated with propofol, 14 (26.42%) with midazolam, and 10 (18.87%) were curarized; whereas 7 patients (13.21%) were lightly sedated with dexmedetomidine. A total of 28 (52.83%) patients received analgesia with Fentanyl. The median Richmond Agitation Sedation Scale (RASS) during the assessments was -3 (q1 = -5; q3 = 0; IQR = 5).

**Survival cumulative probability at 90 days in Hospital and ICU**

Patients who received non-invasive neuromonitoring showed a cumulative probability of survival at 90 days both in Hospital and ICU higher than those who did not received neuromonitoring (**Figure 3 - ESM**).


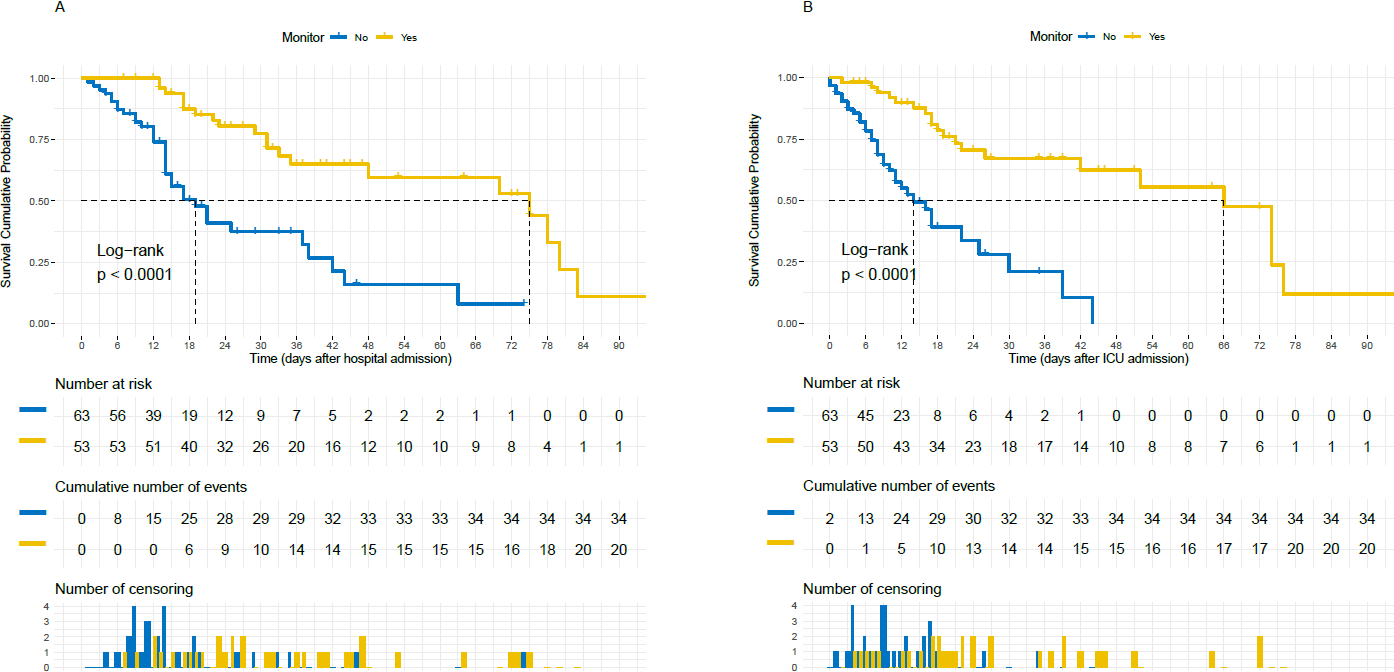


**ESM - Figure 3.** Survival cumulative probability after hospital and ICU admission in the whole population of COVID-19 patients (n = 116), stratifying for not receiving / receiving (No/Yes) non-invasive neuromonitoring.

**ESM - Table 7.** Comparison between normal and high intracranial pressure, evaluated by transcranial Doppler and Optic Nerve Sheath Diameter.

| **Variable** | **Mean ± SD** | **Median** | **q1** | **q3** | **IQR** | **P value** |
| --- | --- | --- | --- | --- | --- | --- |
| Age (years)  High nICP_TCD_ (n = 21)  Normal nICP_TCD_ (n = 28)  High nICP_ONSD_ (n = 10)  Normal nICP_ONSD_ (n = 39) | 65.95 ± 6.82  63.89 ± 9.02  62.70 ± 6.93  65.72 ± 8.19 | 66.00  63.00  64.00  65.00 | 61.00  58.00  60.25  60.00 | 70.00  70.50  67.75  75.00 | 9.00  12.50  7.50  15.00 | 0.380  0.399 |
| BMI (kg/m^2^)  High nICP_TCD_ (n = 21)  Normal nICP_TCD_ (n = 28)  High nICP_ONSD_ (n = 10)  Normal nICP_ONSD_ (n = 39) | 28.33 ± 4.41  26.70 ± 3.26  28.43 ± 5.25  27.31 ± 3.46 | 27.68  26.32  29.84  26.51 | 25.25  24.22  24.07  24.84 | 31.14  29.32  31.14  29.55 | 5.89  5.10  7.08  4.71 | 0.222  0.519 |
| GOS  High nICP_TCD_ (n = 21)  Normal nICP_TCD_ (n = 28)  High nICP_ONSD_ (n = 10)  Normal nICP_ONSD_ (n = 39) | 3.25 ± 0.45  3.19 ± 0.54  3.17 ± 0.41  3.23 ± 0.53 | 3.00  3.00  3.00  3.00 | 0.00  1.00  3.00  3.00 | 3.25  3.25  3.00  3.75 | 0.25  0.25  0.00  0.75 | 0.790  0.751 |
| mRS  High nICP_TCD_ (n = 21)  Normal nICP_TCD_ (n = 28)  High nICP_ONSD_ (n = 10)  Normal nICP_ONSD_ (n = 39) | 3.08 ± 0.67  3.44 ± 0.81  3.50 ± 0.55  3.23 ± 0.81 | 3.00  4.00  3.00  3.00 | 3.00  3.00  3.00  2.00 | 3.25  4.00  4.00  4.00 | 0.25  1.00  1.00  1.00 | 0.150  0.522 |
| PEEP (cm H_2_0)  High nICP_TCD_ (n = 21)  Normal nICP_TCD_ (n = 28)  High nICP_ONSD_ (n = 10)  Normal nICP_ONSD_ (n = 39) | 10.84 ± 2.95  9.13 ± 2.68  11.43 ± 2.46  9.28 v 2.86 | 12.00  9.67  11.33  9.28 | 8.70  7.14  9.44  7.00 | 13.00  10.71  13.25  11.31 | 4.30  3.57  3.81  4.31 | 0.082  0.061 |
| PaCO_2_ (mm Hg)  High nICP_TCD_ (n = 21)  Normal nICP_TCD_ (n = 28)  High nICP_ONSD_ (n = 10)  Normal nICP_ONSD_ (n = 39) | 51.24 ± 15.45  46.59 ± 8.28  51.95 ± 6.70  47.58 ± 12.96 | 47.20  45.40  50.27  43.00 | 39.50  40.81  46.57  39.16 | 57.20  50.53  56.05  53.96 | 17.70  9.72  9.48  14.80 | 0.359  0.031 |
| Hospital stay (days)  High nICP_TCD_ (n = 21)  Normal nICP_TCD_ (n = 28)  High nICP_ONSD_ (n = 10)  Normal nICP_ONSD_ (n = 39) | 38.90 ± 30.34  31.00 ± 19.23  45.00 ± 25.27  36.33 ± 24.70 | 23.00  31.00  40.50  31.00 | 18.00  19.75  23.00  18.00 | 47.00  44.75  66.50  46.00 | 29.00  25.00  43.50  28.00 | 0.691  0.223 |
| ICU stay (days)  High nICP_TCD_ (n = 21)  Normal nICP_TCD_ (n = 28)  High nICP_ONSD_ (n = 10)  Normal nICP_ONSD_ (n = 39) | 32.86 ± 25.55  28.61 ± 20.89  42.30 ± 23.21  28.26 ± 22.28 | 21.00  21.00  38.00  19.00 | 17.00  15.75  21.25  13.50 | 46.00  39.00  65.25  40.50 | 29.00  23.25  44.00  27.00 | 0.721  0.042 |

TCD: transcranial Doppler; ONSD: optic nerve sheath diameter; SD: standard deviation; q1: first quartile; q3: third quartile; IQR: interquartile range; BMI: body mass index; GOS: Glasgow outcome scale; mRS: modified Rankin scale; PEEP: positive end-expiratory pressure; PaCO_2:_ partial pressure of carbon dioxide; ICU: intensive care unit.

**Cox regression models in the patients who underwent non-invasive neuromonitoring**

By evaluating the variable collected in the non-invasive neuromonitoring patients for their potential impact on survival, we found that six variables reached statistical significance satisfying the proportional hazards assumption at the univariate Cox regression [pH (HR: 0.000, p <0.001); PaCO_2_ (HR: 1.103; p <0.001); Na^+^ (HR: 1.102; p = 0.048); PcPs (HR: 1.100, p = 0.023); PEEP (HR: 1.201; p = 0.046); RR (HR: 1.101; p = 0.013)] (**ESM -** **Table 8**). In the subsequent multivariate Cox regression model, only pH (HR: 0.000; p = 0.006) and Na^+^ (HR: 1.300; p = 0.015) returned statistical significance (**ESM -** **Table 8; ESM - Figure 4**). The rank-hazard plot of the multivariate Cox regression model with relative hazard for each covariate is presented in **ESM -** **Figure 5**.

**ESM - Table 8.** The significant variables associated with survival assessed by univariate Cox regression and the output of the subsequent multivariate model, for the patients (n = 53) who underwent non-invasive neuromonitoring.

| **Variable** | **Univariate** | | | | **Multivariate** | | | |
| --- | --- | --- | --- | --- | --- | --- | --- | --- |
|  | **β** | **HR** | **95% CI** | **P value** | **β** | **HR** | **95% CI** | **P value** |
| pH | -13.000 | 0.000 | 0.000 - 0.000 | 0.001 | -22.789 | 0.000 | 0.000 - 0.001 | 0.006 |
| PaCO_2_ | 0.066 | 1.103 | 1.040 - 1.105 | <0.001 | 0.034 | 1.000 | 0.960 - 1.116 | 0.387 |
| Na^+^ | 0.082 | 1.102 | 1.000 - 1.180 | 0.048 | 0.237 | 1.300 | 1.000 - 1.535 | 0.015 |
| Pc/Ps | 0.110 | 1.100 | 1.020 - 1.231 | 0.023 | 0.116 | 1.101 | 0.951 - 1.335 | 0.186 |
| PEEP | 0.161 | 1.201 | 1.000 - 1.372 | 0.046 | -0.282 | 0.750 | 0.530 - 1.072 | 0.116 |
| RR | 0.097 | 1.101 | 1.010 - 1.200 | 0.013 | 0.037 | 1.000 | 0.891 - 1.212 | 0.637 |

Pc/Ps: Pressure control or Pressure support; Na^+^, serum sodium; PEEP: positive end-expiratory pressure; RR: respiratory rate; HR: hazard ratio; CI: confidence interval.


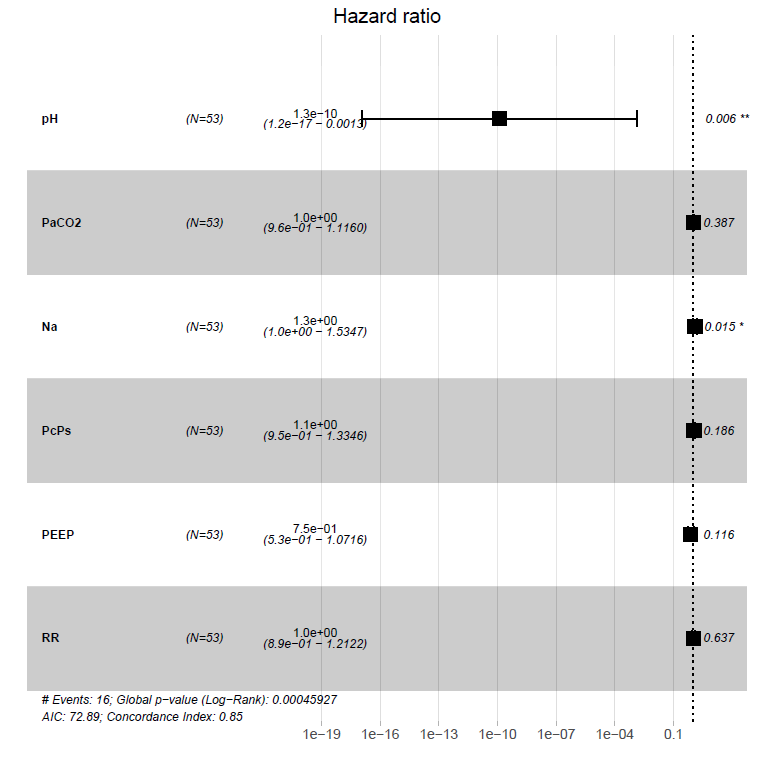


**ESM - Figure 4.** Forest plot of the variables entered in the multivariate Cox regression model, for the patients (n = 53) who underwent non-invasive neuromonitoring.


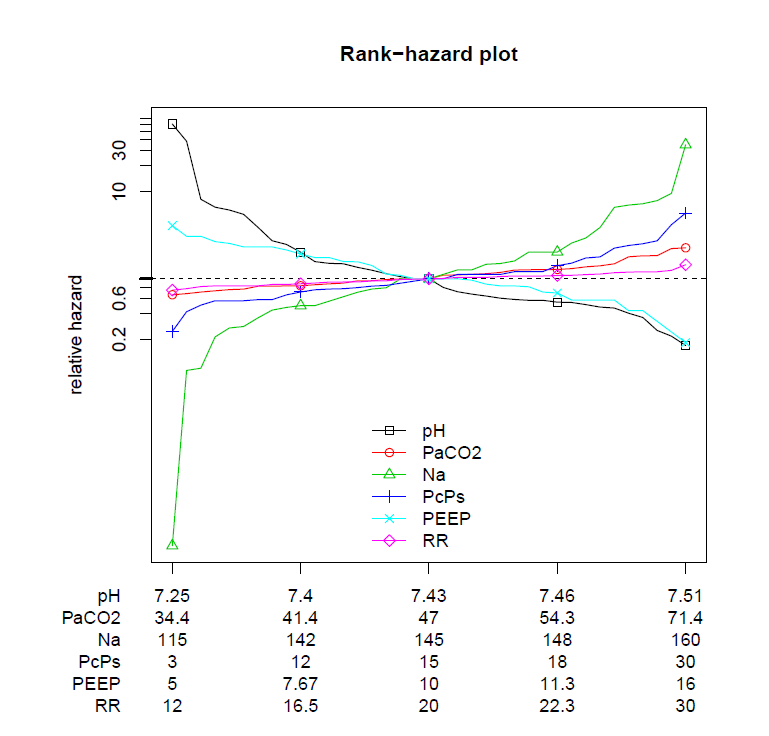


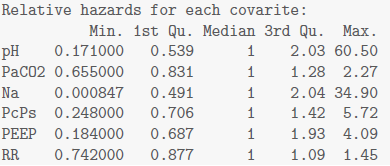


**ESM - Figure 5.** Rank-hazard plot of the variables entered in the multivariate Cox regression model, for the patients (n = 53) who underwent non-invasive neuromonitoring.

**ESM – Case 1**

A 57 year-old man was admitted to our ICU for respiratory failure with confirmed positive for SARS-CoV-2 infection by reverse transcriptase-polymerase chain reaction (RT-PCR) of nasopharyngeal swab specimens. On day 5 he was ventilated with high PEEP (15 cmH20) and with a FiO2 0.60, with PaO2/FiO2= 154. He underwent non invasive ICP monitoring: ONSD measurement showed an increased diameter (0.61 cm).TCCD of the right middle cerebral artery (MCA) demonstrated a very low diastolic flow velocity (FVd) of 18.2 cm/sec, yet a normal systolic flow velocity (FVs) of 130 cm/sec, indicating prevailing cerebral blood flow (CBF) during the systolic phase of the cardiac cycle. On day 11, the patient was weaned from sedation and he experienced hyperkinetic delirium with consequent difficult respiratory weaning. His clinical course was complicated by hospital acquired pneumonia. He was extubated on day 24 and discharged from ICU on day 33.


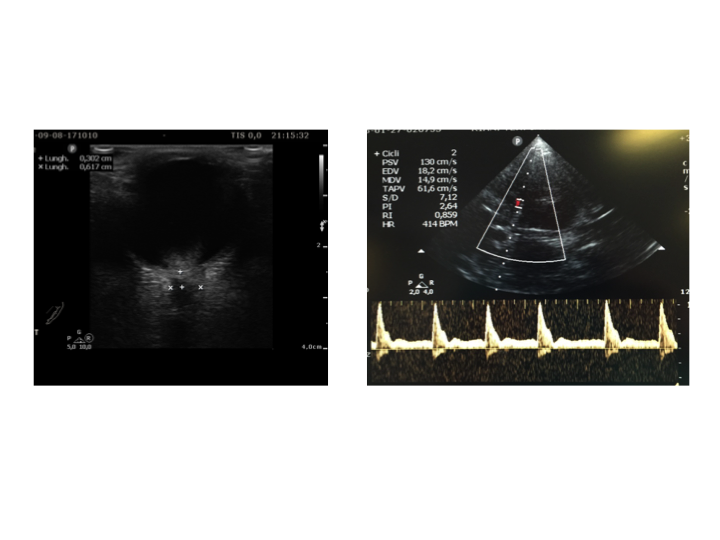

Supplement: Supplementary file 1 [file Data_Sheet_1.docx]
